# Orthodontic traction of impacted maxillary canines using segmented arch mechanics

**DOI:** 10.1590/2177-6709.24.5.079-089.sar

**Published:** 2019

**Authors:** Marco Antonio Schroeder, Daniela Kimaid Schroeder, Jonas Capelli, Diego Junior da Silva Santos

**Affiliations:** 1 Private practice (Rio de Janeiro/RJ, Brazil).; 2 Universidade do Estado do Rio de Janeiro, Faculdade de Odontologia, Departamento de Odontologia Preventiva e Comunitária (Rio de Janeiro/RJ, Brazil).

**Keywords:** Impacted canines, Interceptive orthodontics, Computed tomography, Traction, Impacted teeth.

## Abstract

The principles of orthodontic mechanics strongly influence the success of impacted canine traction. The present study discusses the main imaging exams used for diagnosis and localization of impacted canines, the possible associated etiological factors and the most indicated mechanical solutions.

## RESUMO

Os princípios vetoriais da mecânica ortodôntica têm influência direta no sucesso do tracionamento dos caninos impactados. O objetivo desse artigo é discorrer sobre os possíveis fatores etiológicos associados à impacção dos caninos, os exames de imagem indicados no processo de diagnóstico e localização das unidades retidas, e as melhores soluções mecânicas para o tracionamento desses dentes.

## INTRODUCTION

**Figure 1:**
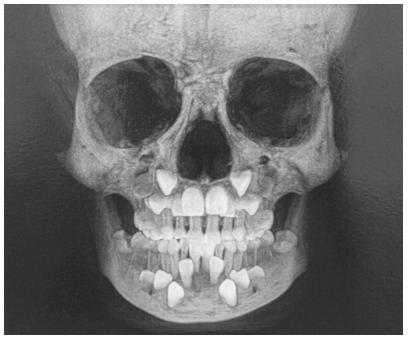
Canines positioned more basally in maxilla and mandible, resulting in longer eruption pathways than those of other teeth to reach final occlusal position.

Maxillary canines begin their calcification at different times from the permanent maxillary molars and central incisors. However, canines take twice the time to reach full eruption, as they move about 22 mm from the orbital floor to their final position, which makes them more vulnerable to environmental factors^1^ (Fig 1).

The eruption of permanent maxillary canines may be explained by the Guidance Theory, according to which canine eruption is guided by permanent lateral incisors. In cases of lateral incisor agenesis or shape anomaly, canines do not find the proper guidances that would enable their normal eruption.^2^

**Figure 2:**
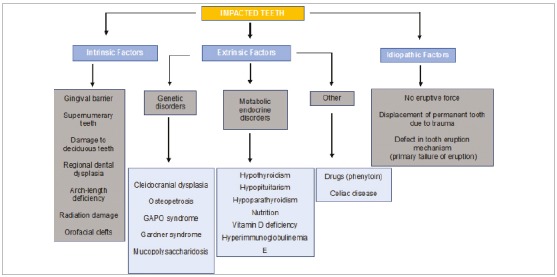
Etiopathogenesis of tooth impaction. Source: based on Kuroda et al.^4^, 2007.

Teeth that stop eruption before emergence are called impacted teeth. The several factors that may block tooth emergence are classified as intrinsic and extrinsic (Fig 2). 

The main intrinsic factors associated with the etiology of tooth impaction are: insufficient space for eruption due to maxillary deficiency; early loss of a deciduous tooth followed by loss of space for the permanent tooth; prolonged retention of deciduous canines; and excessive fibrous gingival tissue, which may also have eruption cysts^3^.

**Figure 3:**
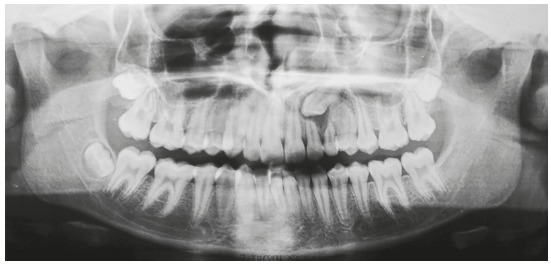
Abnormal root shape of tooth #24 and eruption pathway deviation of tooth #23 resulted in resorption of root apex of tooth #22.

Moreover, premolar rotations may also lead to the obstruction of the canine eruption pathway^1^ (Fig 3).

Some of the extrinsic factors are: syndromes, such as craniofacial dysostosis, also called Crouzon syndrome; metabolic conditions, such as hypothyroidism and hypopituitarism.^4^

## PERMANENT CANINE IMPACTION

Canines, after third molars, are the teeth with the highest prevalence of impaction. Studies have also found that permanent maxillary canine impaction affects women twice as often as men,^5^ and that impaction is more frequently palatal (80-90%) than buccal (10-20%).^6^

The prevalence of canine impaction is 0.8% to 3.6% of the general population. White individuals are more often affected by palatal impaction; among Asian individuals, impaction is usually buccal.^7^ ^-^ ^9^

Buccal canine impaction is associated with skeletal and arch-length discrepancies, although a small percentage of these patients do not have anterior crowding.^10^

Palatal canine displacement is a genetic disorder that may result in palatal impaction, an eruption anomaly that affects 0.2% to 2.3% of orthodontic patients, with a men/women prevalence of 1:3.^11^ ^,^ ^12^

## DIAGNOSIS

**Figure 4:**
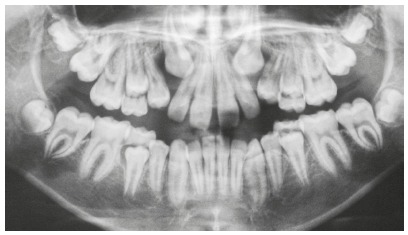
Panoramic radiograph shows displacement of permanent maxillary lateral incisors and first premolars due to impaction of #13 and #23.

Canine impaction may have detrimental consequences, such as root resorption of the lateral incisor and migration of adjacent teeth, followed by dental midline deviation and arch perimeter shortening (Fig 4). 

Due to the severity of the problems associated with permanent canine impaction, a careful and early diagnosis of eruption disorders is mandatory to prevent serious problems and to ensure less treatment trauma. Clinical methods and imaging studies should be used for that. 

Clinical examination and palpation of the alveolar process buccally and palatally are recommended for patients 8 years and older. Clinical examination should take note of the number of erupted teeth, presence of marked lateral incisor displacement or buccal inclination, dentoalveolar arch atresia, loss of space and asymmetric eruption when comparing right and left sides of the dental arch. These findings should be analyzed together with the patient’s age. This analysis should be individual and according to the development of each patient. This strategy avoids unnecessary radiographs.

In order to help orthodontists, several studies have evaluated variables that may be associated with permanent canine impaction, such as position and development, overlapping with adjacent incisor roots, root resorption and anomalies, and linear and angular measurements using radiographs and CT scans.^13^ ^-^ ^15^

Panoramic radiographs are a routine diagnostic tool in orthodontists’ offices. Ericson and Kurol^16^ used them to validate measurements to diagnose and predict palatal canine impaction.

**Figure 5:**
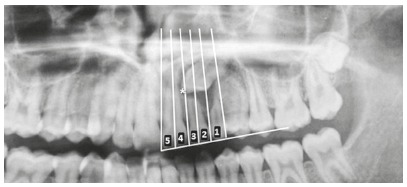
According to Ericson and Kurol,^16^ permanent maxillary canines are palatally impacted when canine crown is between sectors 2 and 5.

**Figure 6:**
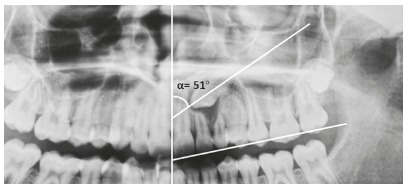
According to Ericson and Kurol,^16^ an angle α ≥ 15° is suggestive of canine impaction.

The measurements established by those authors on panoramic radiographs are (Figs 5 and 6):

1. Alpha angle (α): mesial tipping of permanent canine crown from midline.
2. Distance (d): distance from permanent canine cusp tip to occlusal plane.
3. Sector: mesial position of the canine crown in relation to central and lateral incisors.

There are five sectors: sector 1 indicates the position of the impacted canine crown posterior to the distal surface of the permanent lateral incisor root, and sector 5 corresponds to the mesial half of the permanent central incisor. Canines with α ≥15^o^ between sectors 2 and 5 and an intraosseous position in the palate are classified as palatally impacted^11^.

However, the diagnostic accuracy of identification of impacted canine position and adjacent structures may be not reliable because of the deficiencies of two-dimensional radiographic images, such as distortions and lack of sharpness, which may increase the risk of an incorrect interpretation.^16^ ^,^ ^17^

An inaccurate diagnosis and treatment approach may lead to complications during patient development and deterioration of the impaction. The most common complication is root resorption of adjacent tooth roots. Resorption may affect the lateral incisor, the central incisor, or both at the same time. There are usually no clinical symptoms, not even when the pulp is affected. The risk of resorption is greater among female patients when they already have more than half of the canine root, when the canine is beyond the center of the lateral incisor, and when canine tipping exceeds 25^o^ from the midline. When root resorption is diagnosed at an advanced stage, treatment is more difficult and may require the extraction of the lateral incisor.^17^ ^,^ ^18^

In 80% of the cases, the initial diagnosis may be made using radiographs. When canines and lateral incisors are overlapping, it is not possible to determine whether there is resorption. When the lamina dura of the lateral incisor is not detected, when the root outline is irregular, or in other conditions that may affect treatment planning, CT is indicated.^18^

## TREATMENT

The purpose of interceptive treatment of canine impaction is to increase the chances of spontaneous eruption of these teeth. It may be conducted in several ways: extraction of deciduous canines; extraction of deciduous canines and first premolars; increase of space in the region of the arch close to impaction; extraction of deciduous canines with or without use of extraoral appliance;^19^ extraction of first premolar as part of serial extraction; and extraction of lateral incisor with abnormal shape.^20^

Extraction of deciduous canines without root resorption in children aged 9 to 12 years may correct palatal ectopic position in 80% of the cases. Seventy-four percent of the cases may be corrected when the canine cusp is beyond the center of the lateral incisor on radiographs. This percentage increases to 91% when the canine crown is distal to the center of the lateral incisor.^18^

The result is less favorable in the following cases: older patients; lack of space in the dental arch; permanent canine with complete root formation; and horizontal and superior position to the alveolar process.

Clinical and radiographic follow-up should be conducted every six months to evaluate possible changes in the pattern of eruption. If there are no changes after 12 months, it is necessary to change treatment strategy.

**Figure 7:**
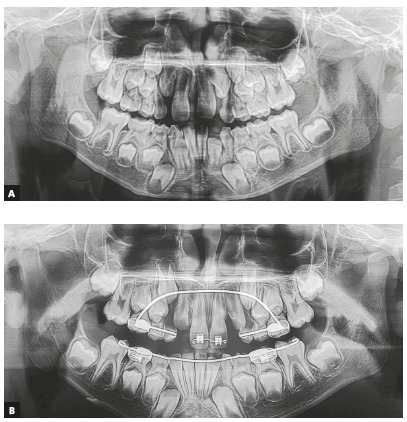
A) Panoramic radiograph showing lack of maxillary arch space. B) After four months of retention, Hyrax expander was replaced with transpalatal arch soldered to bands cemented to deciduous maxillary second molars, extending to deciduous canines. A lingual archwire was used for expansion of mandibular arch.

In patients that undergo maxillary expansion, retention should be kept for a longer time. First, the Hyrax or Hass appliance should be kept as a retainer for four to six months. After that, it should be replaced with a transpalatal arch extending to teeth #53 and #63, when they are present, or to the deciduous first molars (#54 and #64), when extraction of deciduous canines is requested. For greater maxillary arch expansion, the mandibular arch may also have to be expanded, in some cases (Fig 7). 

**Figure 8:**
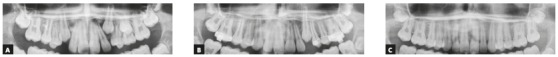
A) Tooth #23 impaction, due to lack of space. B) Extraoral cervical appliance used to obtain space for teeth #13 and #23. C) Posttreatment panoramic radiograph showing teeth #13 and #23 in line of occlusion.

Another resource to gain space when there is sagittal, but no transverse deficiency is the use of an extraoral cervical appliance. This procedure may also be associated with the extraction of the deciduous maxillary canines (Fig 8). 

**Figure 9:**
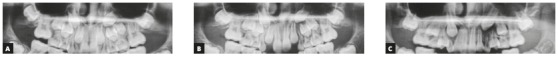
Sequence of panoramic radiographs showing patient from 6 to 9 years of age: A) 6 years, B) 7 years, C) 9 years. Direction of canine eruption axis remained the same even after space was obtained.

Sometimes, there is no spontaneous correction of canine position because of excessive intraosseous tipping even after space has been expanded. In these cases, orthodontic forced eruption should be used (Fig 9).

The best clinical management option is, undoubtedly, interception during mixed dentition, when the root is not yet fully formed. However, if impaction cannot be prevented, traction may be the best option, as the maxillary canine has a fundamental role both in the patient’s facial aesthetics and, mainly, in mastication, during the lateral mandibular movements.

**Figure 10:**
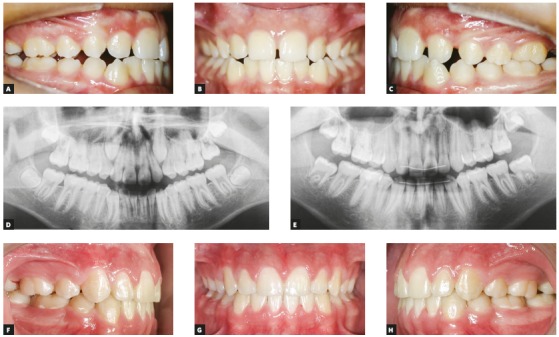
A-D) Pretreatment intraoral photographs and panoramic radiograph. E-H) Postreatment intraoral photographs and panoramic radiographs. Teeth #14, #24, #35 and #45 were extracted.

When canines are impacted because of lack of space, although their position is favorable for eruption, the best alternative may be the extraction of the first premolar (Fig 10). In some cases, space gains without extractions may be an option, but that will depend on biological limits, the risk to neighboring structures, patient tolerance to a longer treatment and patient compliance.

When the canine does not erupt spontaneously, surgical exposure and orthodontic repositioning are predictable options with good results in most cases. This type of treatment includes a multidisciplinary approach that should count on the interaction between oral and maxillofacial surgeons, periodontists and orthodontists.

**Figure 11:**
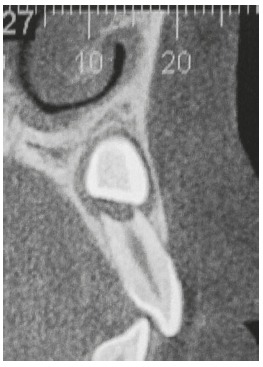
Oblique sagittal slice identifying canine buccolingual position and its amount of overlying bone.

The amount of bone to be removed during crown exposure is determined by the position of the impacted tooth and the surgical method of exposure for device bonding to the crown (Fig 11). Excessive bone removal may increase chances of loss of insertion and of possible damage to the root of the adjacent tooth.^21^ An informed consent form is recommended to clarify to the patient that there might be other complications associated with traction, such as loss of pulp sensitivity, resorption of root surface of the tooth undergoing traction, changes in color, impossibility of movement due to ankylosis and changes in the position of the tooth that has been moved, after the treatment is completed (relapse). 

**Figure 12:**
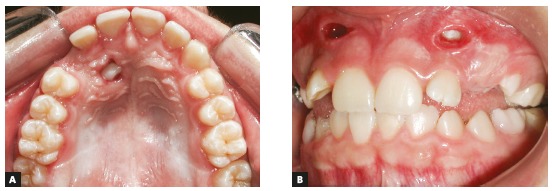
Examples of impacted canine exposure using open surgical technique. A) Palatal exposure, and B) Labial exposure.

**Figure 13:**
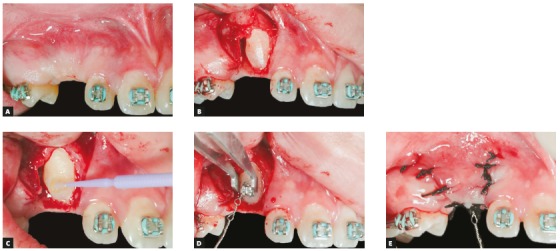
Example of impacted canine exposure using closed surgical technique. A) Pretreatment photograph, B) incision is made and gingival flap is raised, C) enamel surface etching, D) device bonding, E) gingival flap is repositioned and sutured.

Canine traction, after either open or closed exposure, is one of several surgical procedures described in the literature. In the open technique, a circular section of the mucosa and alveolar bone is removed (Fig 12). In the closed procedure, the device is bonded during the exposure of the impacted tooth and, after that, tissues are repositioned and sutured to cover it (Fig 13).^22^

Several types of incisions have been suggested for canine exposure. In general, it should provide adequate access to the crown and efficient bleeding control during device bonding. In addition, the type of flap should ensure good blood supply. In buccal incision, the ideal procedure is to have a flap that includes keratinized gingiva and that is repositioned and sutured apically to prevent gingival recession.

Surgical exposure in adolescents leads to spontaneous eruption of the canine in 65% of the cases; however, treatment time is longer, and the eruption pathway cannot be controlled.^24^

## SEGMENTED ARCHWIRE MECHANICS

**Figure 14:**
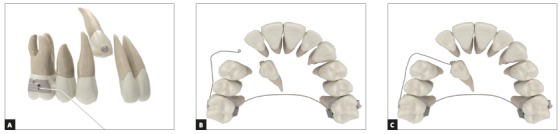
A) Lateral view of segmented archwire passively attached to accessory tube on first molar. B) Occlusal view of another cantilever. Transpalatal arch used to minimize reaction forces of anchorage unit. C) A resultant force is generated when cantilever is attached to canine accessory and moves canine labially.

Segmented archwires for impacted canine traction have a simple design: a wire that connects an anchorage device, which may be a bracket or a tube, to the tooth to undergo traction. The force system is determined by folding the wire segment, and the direction of the resulting force is easy to be clinically identified (Fig 14).

**Figure 15:**
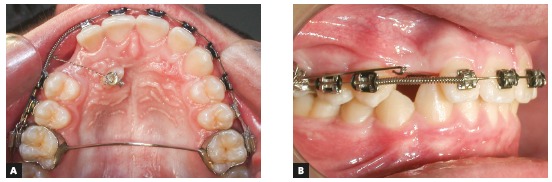
A) Upper occlusal photograph and B) right side intraoral photo of impacted canine traction using 0.019 x 0.025-in stainless steel cantilever. B) Omega loop is attached and tied to the tube using metal ligature to stabilize cantilever.

The longer the distance between the two points, the lower its load/deflection rate, which, therefore, produces relatively constant forces and momentum as the teeth move into the desired position. Stability and good adaptation of the segmented archwires are achieved by using a rectangular wire. The resulting force is dependent on the amount of activation, that is, the distance from the point of anchorage to the tooth to be moved, as well as on archwire alloy and thickness. For longer segmented archwires, the recommendation is to use 0.019 x 0.025-in stainless steel wires, and for the shorter ones, 0.018 x 0.025-in TMA. Another stability factor to ensure safe attachment of wire to the tube is to use an omega loop to be tied to the tube (Fig 15).

Unwanted side effects of the anchorage unit on teeth may be mitigated by using auxiliary devices, such as a transpalatal arch or Nance button. The choice of which type of auxiliary device to use depends on the resultant force. If the objective is to move the canine vertically (extrusion) or sagittally (distalization), a Nance button should be used to avoid intrusion, tipping and mesial displacement of the molar crown. If the canine is to be moved buccally, a transpalatal arch is enough for auxiliary anchorage, which prevents molar rotation. 

**Figure 16:**
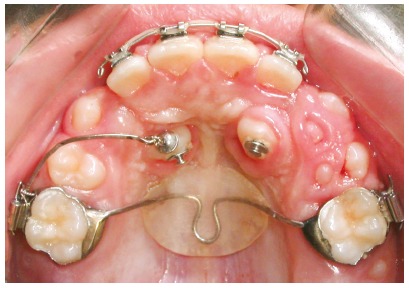
Application of traction force on canine.

In addition to the auxiliary device, a continuous archwire, attached to the brackets, may ensure that anchorage is reinforced. For that purpose, the device bonded to the molar has to be a double tube. To avoid overload of the anchorage during vertical traction of two canines at the same time, force should be applied in turns instead of simultaneously (Fig 16).

**Figure 17:**
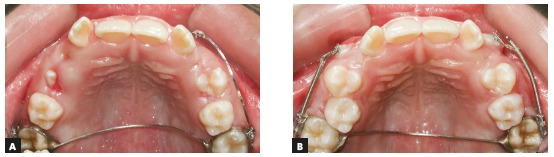
A) Start of canine traction, B) Lack of adequate anchorage led to mesialization and intrusion of teeth #16 and #26.

Lack of attention to anchorage in canine traction using segmented archwires may lead to side effects that affect anchorage teeth, which makes finishing the case more difficult and increases treatment time (Fig 17).

**Figure 18:**
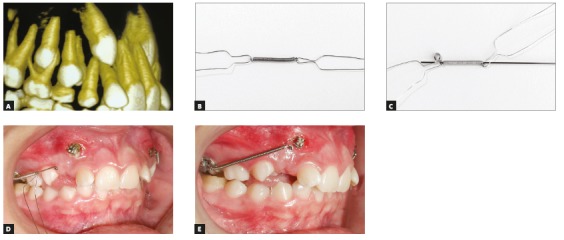
A) Position of canine in relation to lateral incisor root. B, C) Material used for traction: metal ligatures, open nickel-titanium coil spring and 0.018 x 0.025-in stainless steel segmented archwire. D, E) Spring activation using segmented archwire as guidance.

When the position of the canine crown is buccal to the lateral incisor and over its root, it should usually be moved buccally to avoid touching the lateral incisor root. After that, it should be distalized and extruded. These three movements may be achieved at the same time if a closed coil spring is used around the segmented archwire. The force is directed by the archwire segment; the distalization force is generated by tying the coil spring at the distal end, at the point of anchorage, while the mesial end is activated and attached to the canine (Fig 18).

**Figure 19:**
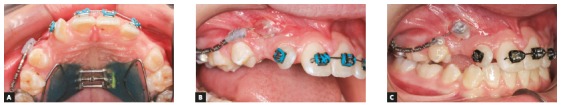
A) Patient in mixed dentition phase underwent maxillary arch expansion. Expander was used as anchorage for segmented archwire with spring, reinforced with anchorage unit to ensure movement in three planes. B) Labial and distal movement by segmented archwire and closed coil activation. C) Simultaneous extrusive and distal movement.

The complexity of these movements requires the use of a reinforced anchorage unit. When there is risk of collapse between lateral incisor root and canine crown, the lateral incisor should not be bonded and moved during the alignment and leveling phase (Fig 19).

**Figure 20:**
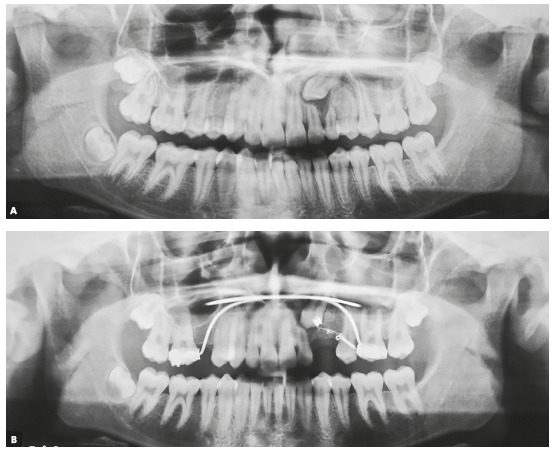
A) Pretreatment panoramic radiograph showing tooth #23 impaction and #22 root resorption. B) Intermediary phase panoramic radiograph showing absence of teeth #15 and #24, transpalatal arch for anchorage and direction of canine traction.

**Figure 21:**
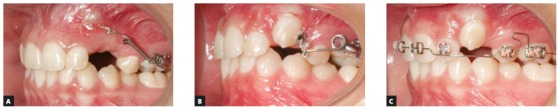
A) Segmented archwire ensures parallel action of force distally. B) Device bonded distally on crown of tooth #23. C) Alignment and leveling without including tooth #22, to avoid further deterioration of root resorption.

**Figure 22:**
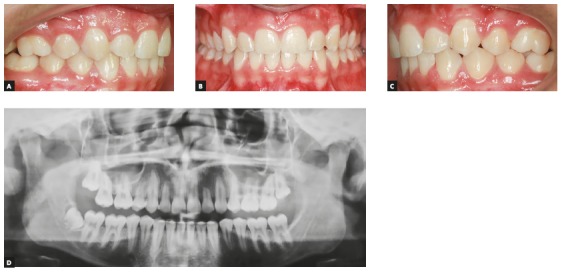
A, B, C) Intraoral posttreatment photographs. D) Posttreatment panoramic radiographic shows root length preservation of tooth #22.

If root resorption is already affecting the lateral incisor, distalization mechanics should be precise, and the lateral incisor should not receive orthodontic forces to avoid the further deterioration of resorption (Figs 20 to 22). 

## CONCLUSIONS

There are several mechanical systems for impacted canine traction, and segmented archwires are probably the most adequate for it. They are easy to manufacture, their cost is low, they have good stability and adapt to different cases, which makes it possible to apply light (25 to 100 grams) and constant forces, with no side effects on adjacent teeth. The direction of traction can be controlled, and other mechanics may be used at the same time.

The use of a coil spring in the segmented archwire is a technique that improves the chances of success of buccal traction of impacted canines that are positioned mesially to their normal axis of eruption. For that purpose, we recommend that careful attention be paid to segmented archwire thickness and type of alloy, force applied to the coil spring, and type of anchorage for each situation. 
